# Specialized Roles for Actin in Osteoclasts: Unanswered Questions and Therapeutic Opportunities

**DOI:** 10.3390/biom9010017

**Published:** 2019-01-09

**Authors:** Guanghong Han, Jian Zuo, Lexie Shannon Holliday

**Affiliations:** 1 Department of Oral Geriatrics, School and Hospital of Stomatology, Jilin University, Changchun 130021, China; ghan@dental.ufl.edu; 2 Department of Orthodontics, College of Dentistry, University of Florida, Gainesville, FL 32610, USA; jzuo@dental.ufl.edu; 3 Department of Anatomy & Cell Biology, College of Dentistry, University of Florida, Gainesville, FL 32610, USA

**Keywords:** actin, microfilament, vacuolar H^+^-ATPase, V-ATPase, anti-resorptives, bone, bone remodeling, actin polymerization

## Abstract

Osteoclasts are cells of the hematopoietic lineage that are specialized to resorb bone. In osteoclasts, the actin cytoskeleton engages in at least two unusual activities that are required for resorption. First, microfilaments form a dynamic and structurally elaborate actin ring. Second, microfilaments bind vacuolar H^+^-ATPase (V-ATPase) and are involved in forming the V-ATPase-rich ruffled plasma membrane. The current review examines these two specialized functions with emphasis on the identification of new therapeutic opportunities. The actin ring is composed of substructures called podosomes that are interwoven to form a cohesive superstructure. Studies examining the regulation of the formation of actin rings and its constituent proteins are reviewed. Areas where there are gaps in the knowledge are highlighted. Microfilaments directly interact with the V-ATPase through an actin binding site in the B2-subunit of V-ATPase. This binding interaction is required for ruffled membrane formation. Recent studies show that an inhibitor of the interaction blocks bone resorption in pre-clinical animal models, including a model of post-menopausal osteoporosis. Because the unusual actin-based resorption complex is unique to osteoclasts and essential for bone resorption, it is likely that deeper understanding of its underlying mechanisms will lead to new approaches to treat bone disease.

## 1. Introduction

**Figure 1:**
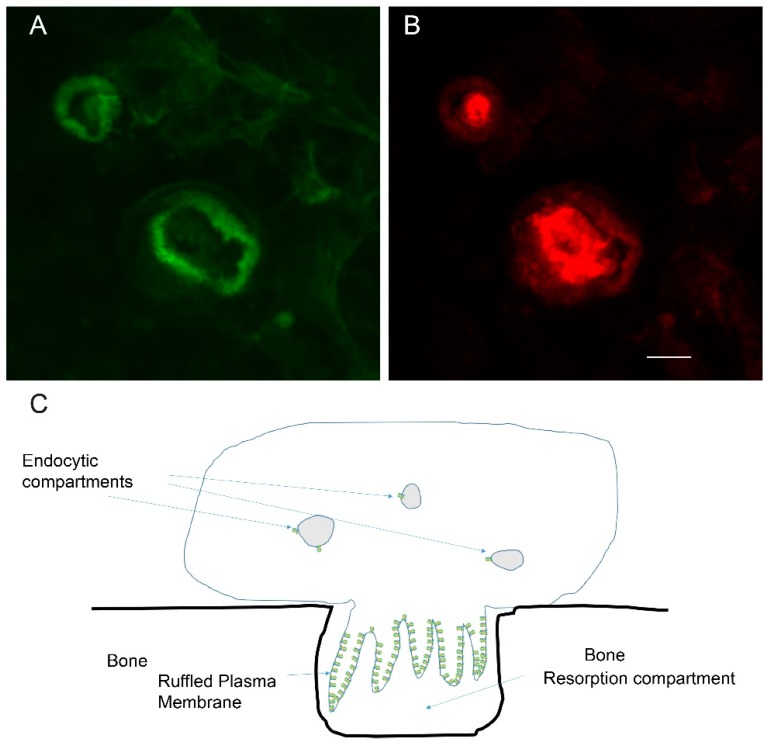
Osteoclasts are specialized cells that can resorb mineralized matrix. To do this they form a resorption compartment that depends on generation of an actin ring and ruffled plasma membrane; (**A**) Confocal micrograph of resorbing osteoclasts on a bone slice stained with phalloidin, which detects filamentous actin, and pseudocolored green. Two actin rings are shown in the field of view; (**B**) The same cells as in ‘A’ are stained (pseudocolored red) with an antibody that binds the E-subunit of the V-ATPase, a component of the ruffled plasma membrane. The vacuolar H^+^-ATPase (V-ATPase) is responsible for pumping protons to acidify an extracellular resorption compartment. The scale bar for ‘A’ and ‘B’ is 5 µm; (**C**) Schematic of a resorbing osteoclast. The ruffled membrane is packed with many V-ATPases (indicated in green). In contrast, endocytic compartments in osteoclasts and other cells require only a few V-ATPases to acidify the compartment. The actin ring, depicted in cross section, is based on actin filaments undergoing rapid polymerization at the cytosolic face of the plasma membrane, and depolymerization toward the center of the cell. The plasma membrane abutted by the actin ring conforms tightly with the bone, creating the extracellular resorption compartment.

The osteoclast is a cell type that is specialized for invasion through mineralized matrix [1,2]. To accomplish this feat, osteoclasts form a transient resorption complex, which is composed of actin rings and a ruffled plasma membrane, as shown in Figure 1. The ring is associated with a “sealing zone”, a region of tight association between the plasma membrane of the osteoclast and the bone that seals off an external resorption compartment. The actin ring surrounds a plasma membrane domain called the ruffled membrane (or ruffled border) due to brush border like ruffles that are packed with vacuolar H^+^-ATPase (V-ATPase) [3,4]. V-ATPases pump protons into the resorption compartment, thereby lowering the pH, which solubilizes the bone mineral and provides an acidic environment for the degradation of the bone’s organic matrix by the acid cysteine proteinase, cathepsin K, which is secreted by osteoclasts [5,6]. 

The actin ring was first described in detail by an elegant transmission electron microscopy study of calvarial osteoclasts [7], which showed the overall “bottle brush” appearance of the individual podosomes, and the interconnections between distinct podosomes by a loose network of microfilaments. The identity of the microfilaments was confirmed by making use of labeling with myosin S1. This study, performed when the characterization of the actin cytoskeleton was still in its infancy, provided a solid structural basis for the research that has followed. 

A surprising early view of the crucial nature and regulation of the actin ring in bone resorption came when the tyrosine kinase pp60c-src was knocked out in mice [8]. The severe pathologies detected were secondary to defects in bone resorption. These included failure of tooth eruption, growth reduction, and cranial malformation. The brain and platelets, where c-src is most highly expressed, and other tissue and organ systems did not reveal abnormalities. This was presumably due to functional overlap with related tyrosine kinases. In vitro and in vivo studies showed that osteoclasts in the pp60 c-src knockout animal did not make normal numbers of ruffled membranes and actin rings, and had reduced ability to resorb bone [8,9,10]. It is worth noting that osteoclasts isolated from the c-src knockout animals were able to form some normal pits in resorption assays, but the number was greatly reduced [9]. More recent studies show that c-src regulates the formation, structure, life span, and rate of actin polymerization in podosomes and in the actin cloud that surrounds podosomes [10].

Actin rings of osteoclasts were shown to be dynamic, going through rounds of actin ring formation and resorption, followed by periods of movement without resorption or actin ring formation [11,12,13]. It also emerged that an array of “focal adhesion” proteins were concentrated in the area of the actin ring [11,14,15,16,17]. These proteins are associated with integrins, suggesting that actin rings are controlled and regulated by integrin-based signaling. Many additional pieces of evidence that this idea is correct emerged, including the use of integrin inhibitors to block bone resorption [18,19,20,21,22,23,24]. Kindlin-3, a blood cell specific activator of β1, β2, and β3 integrins, which are all found in osteoclasts, results in osteopetrosis in mice associated with dysfunctional actin ring formation [25]. These data suggest that multiple classes of integrins are likely involved in the formation of the actin ring and organization of the actin ring. Knockout or certain mutations of αVβ3 integrin results in skeletal defects [26]. αVβ3 ligands (RGD-sequences) trigger the activation of osteoclasts. An important route for these ligands to be uncovered is through the action of interstitial collagenase released by osteoblasts or lining cells associated with the bone, which cleaves type I collagen, which in turn exposes Arginine, Glycine, Aspartate (RGD)-sequences that are cryptic in native collagen but exposed when collagen is gelatinized by cleavage [27].

It was also demonstrated that V-ATPase is the proton pump required for acidification in the resorption compartment [3,4]. This finding emphasized the unusual nature of osteoclasts. In most cells, V-ATPases are expressed at very low levels and are not found in the plasma membrane. In contrast, osteoclasts, along with intercalated cells and proximal tubule cells in the kidney, and cells from a few other locations, express very large amounts of V-ATPase and it is localized in a specialized domain of the plasma membrane when the cells are activated [28]. 

## 2. Actin Dynamics in the Actin Ring

Podosomes are visible by differential interference light microscopy and were studied in living osteoclasts. They appear and disappear during a period of a few minutes [29,30]. Studies from the Jurdic lab examined osteoclasts on glass (inactive) [31] and on hydroxyapatite (resorbing) [32] expressing green fluorescent protein-actin in the osteoclasts and used fluorescent recovery after photobleaching to examine the dynamics of the osteoclast actin cytoskeleton. The podosomes of osteoclasts on glass coverslips differ in size, and in turnover rate of actin filaments, from those in osteoclasts on hydroxyapatite [31,32]. In both cases, recovery of actin in a photobleached area took around 30 s. However, because podosomes in the actin rings of resorbing cells are much larger (extending 4 µm from the plasma membrane compared to 1 µm in inactive osteoclasts) the fact that they recover after photobleaching in the same time suggests much higher rates of actin turnover occur in actin rings of resorbing osteoclasts [32]. These results make clear that to understand the actin rings of osteoclasts requires understanding of the means by which actin polymerization and depolymerization are controlled. 

Actin dynamics is associated with ATP hydrolysis [33]. The dynamics of the actin ring microfilaments together with the ATP required for proton pumping by the V-ATPase requires a larger energy expenditure by the resorbing osteoclast compared with most cells. To produce this, three specialized mechanisms are in place. First, osteoclasts express large numbers of mitochondria [34]. A recent study showed that mitochondria are involved in positioning of the actin ring through a pathway involving PGC1β [35]. Second, osteoclasts rely on carbonic anhydrase 2 to convert carbon dioxide created by mitochondrial metabolism to protons and bicarbonate ions. The protons offset those lost while acidifying the extracellular resorption compartment, while the bicarbonate is exchanged for chloride ions through a plasma membrane chloride/bicarbonate exchanger [36]. Finally, in osteoclasts, aldolase and other glycolytic enzymes bind directly to the V-ATPase, providing a mechanism to both locally generate ATP for proton pumping and protons, which are a byproduct of glycolysis [37,38]. The energy metabolism changes in osteoclasts as they differentiate, shifting from primarily oxidative phosphorylation in immature osteoclasts, to glycolysis as osteoclast resorb [39].

## 3. Mechanisms Regulating Microfilament Dynamics in the Actin Ring

### 3.1. Actin-Related (Arp)-2/3 Complex and the Osteoclast Actin Ring and Resorption Compartment

Actin is one of the most abundant proteins in eukaryotic cells [33]. It usually represents between 5% and 15% of the protein bulk weight. Typically, half of the actin is polymerized into microfilaments (actin filaments, F-actin) and the other half is unpolymerized actin (G-actin) [40]. The critical concentration of actin in a salt solution resembling the cytosol is around 0.1 µM. In cells, the concentration of G-actin is typically 10–50 µM. Cells are thought to maintain high G-actin concentrations by two strategies. The first is by an actin monomer being transiently bound to proteins like thymosin B4, profilin, or cofilin [33]. Actobindin transiently traps an actin dimer preventing it from polymerizing [41]. The binding of actin to these sequestering proteins prevents spontaneous polymerization, and in some cases, addition to pre-existing filaments. The second is by proteins that block the ends of microfilaments, blocking the addition of additional actin. Due to these strategies, little or no actin polymerizes spontaneously in cells and polymerization is tightly regulated. A result of the high unpolymerized actin concentration in the cytosol is that when polymerization is triggered, it can occur very rapidly [32]. 

**Figure 2:**
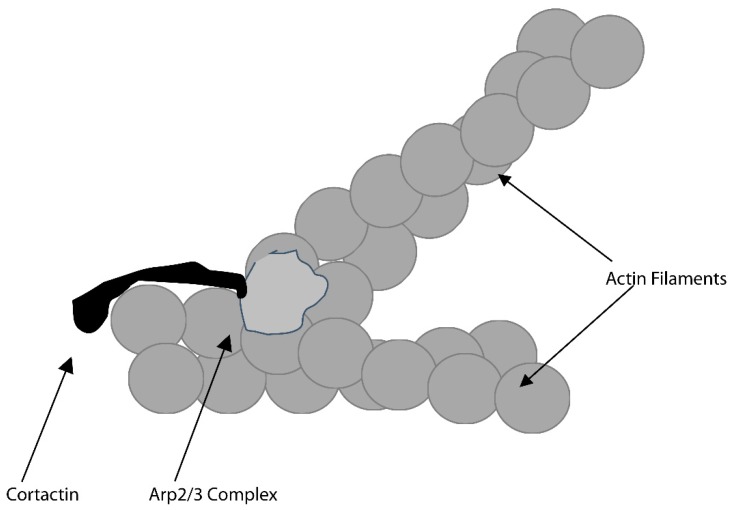
A protein complex that includes cortactin and the actin-related protein (Arp) 2/3 complex triggers actin polymerization that forms new filaments at a roughly 70-degree angle from the “mother” filament. This likely forms a base structure of the actin ring.

There are two general mechanisms that control actin polymerization. One requires the actin-related protein (Arp) 2/3 complex; the second relies on a class of proteins called formins [42]. The Arp2/3 complex is composed of seven proteins. These include two members of the actin superfamily, Arp2 and Arp3. The complex can be either inactive or active. When activated Arp2 and Arp3 are positioned in the complex to form a dimer that acts like a stable slow-growing end of an actin filament. Monomers can add onto the fast-growing end, until that end is bound by a protein that “caps” the end, preventing additional elongation. Such proteins include gelsolin and capZ. Cortactin, a major substrate for pp60c-src [43] interacts with the Arp2/3 complex and neural Wiskott–Aldrich Protein (n-WASP) [44]. It is able to bind to the side of an actin filament and recruit the Arp2/3 complex and n-WASP, which interact with Arp2/3 complex to stimulate its ability to trigger actin polymerization. The Arp2/3 complex/n-WASP/cortactin complex triggers polymerization of a “daughter” actin filament at roughly a 70-degree angle from the preexisting filament, as shown in Figure 2. This led to the hypothesis that the Arp2/3 complex may be crucial for regulating the polymerization associated with actin rings and that collaboration between the Arp2/3 complex, cortactin and n-WASP may be crucial for the formation of the “bottle brush” like structure of the podosomes. 

Evidence soon appeared that supported this idea. In 2004, the distribution of the Arp2/3 in osteoclasts was reported by labeling using anti-Arp2 and anti-Arp3 antibodies and RNA interference was used to knockdown these proteins [45]. This resulted in dissolution of actin rings and podosomes, even though the cells survived for a significant period of time with the reduced level of Arp2/3 complex. This may have been because sufficient amounts of the complex remained to perform necessary housekeeping functions, and/or actin polymerization triggered by other means (presumably formins) was sufficient for housekeeping functions. 

Cortactin, which is not normally found in hematopoietic cells [46], is strongly upregulated during osteoclast formation [14]. This protein is a major substrate for c-src [43]. As expected, it is crucial for actin ring formation. In 2006, three groups showed that knockdown of cortactin led to osteoclasts that did not form actin rings, although they maintained the more typical lamellipods and remained motile [47,48,49]. The tyrosine phosphorylation sites were found to be important in regulating the amount of polymerization in the actin rings. 

Neural-Wiskott-Aldrich syndrome protein (n-WASP) is a member of the WASP family of proteins, which are involved in activating the Arp2/3 complex to trigger actin polymerization [50]. Activation of n-WASP and other members of the family can occur through the small GTPases, CDC42 and Rac, by phosphatidylinositol-4,5-bisphosphate, by binding to various adapter proteins and by direct tyrosine phosphorylation [50]. N-WASP has not been knocked out from osteoclasts. Osteoclasts from WASP knockouts could resorb bone, but displayed abnormalities in cytoskeletal organization [51]. Wiskott–Aldrich and Scar Homolog (WASH), another family member, would seem to be a reasonable candidate for having a role in osteoclasts. In the slime mold amoeba, *Dictyostelium discoideum*, WASH was reported to interact with V-ATPase and to be involved in its vesicle trafficking [52]. Members of the WASH protein complex were shown to be upregulated during osteoclastogenesis [53]. However, knockouts of WASH in hematopoietic cells in mice do not result in bone defects [54]. Taken together, the accumulated data support the idea that the Arp2/3 complex, in conjunction with cortactin and members of the WASP family, are likely directly responsible for actin polymerization in actin rings. There may be overlap of function among the members of the WASP family, but the details are not clear. For example, are the cytoskeletal defects observed in WASP knockout osteoclasts due to a specific activity of WASP versus other family, or are they due to a reduction in the ability to trigger actin polymerization which could possibly be rescued by increased expression by other family members? A number of other members of the WASP family exist including SCAR/WAVE1, WHAMM (WASP homolog associated with actin, membranes, and microtubules), and JMYWAML (WASP and MIM like) and WAWH (WASP without WH1 domain) [50]. It is not known whether any of these proteins are expressed in osteoclasts or are upregulated during the process of osteoclastogenesis. 

WASP interacting protein (WIP) was shown to be vital for podosome formation [55]. WIP is known to interact with WASP and cortactin [56,57]. 

Data on formins in osteoclasts are surprisingly limited. One publication implicated formin mDia2 in establishing the location of podosomes [58]. Two more recent reports suggest that formin FRL1 (FMNL1) is an essential component of podosomes in macrophages, but to date the role of this protein in osteoclasts has not been examined [59,60].

Both gelsolin and cofilin have been implicated in the actin depolymerization that is required to maintain the dynamic actin rings [61,62]. Cofilin is regulated by Lim kinase 1. Mice in which Lim kinase 1 is knocked out display osteoporosis [63]. This could be due to alterations in the osteoclast or osteoblast cytoskeleton, or perhaps through other regulation. A recent study reported that cofilin interacts with cortactin, and that cofilin is required for the podosomal patterning required for actin ring formation and bone resorption [64]. Cofilin binds actin monomers and prevents polymerization, and also binds the sides of actin filaments and when activated in response to regulation by LIM kinase 1, triggers breaks in the filament leading rapid depolymerization. 

Gelsolin has also been implicated in actin ring formation [61]. Gelsolin, which can cap the fast-growing ends of filaments, and also cause filaments to break and depolymerize [65], was initially found to be associated with src-dependent phosphoinositide 3-kinase regulation of actin rings [66]. Knockout of gelsolin in mice results in osteopetrosis and stronger bones, which resulted from decreased osteoclast bone resorption and abnormal podosome and actin ring formation. 

### 3.2. Triggering of Actin Ring Formation-Integrins, CD44, Bone Mineral, Other Candidates

**Figure 3:**
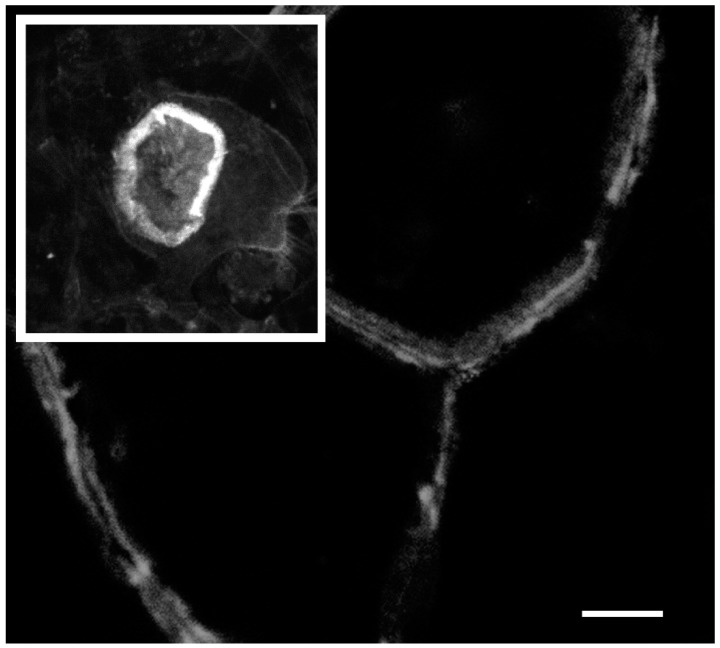
The actin ring of osteoclast resorbing bone (inset) is thicker, larger, and more dynamic than the actin belt of non-resorbing osteoclasts on a glass coverslip. In the main picture, several large osteoclasts form an almost epithelial like sheet with the actin belt of the contacting cells forming side-by-side. The scale bar is 10 µm for the inset picture and 25 µm for the main picture.

As described above, the size intensity and organization of podosomes varies dramatically between osteoclasts on plastic or glass substrates compared with osteoclasts on bone or dentin [31,32], as shown in Figure 3. However, the precise requirements for osteoclast activation are elusive. In cultures containing both osteoclasts and osteoblasts, osteoclast activation was triggered by the activity of interstitial collagenase, which at least in part functioned by exposing RGD-sequences by cleaving and thus denaturing type I collagen, thereby exposing cryptic RGD-sequences [27,67]. More recently, a study utilizing bone biomimetics showed that osteopontin, an RGD-containing protein, stimulated bone resorption by mixed cultures [68]. To construct bone biomimetics, osteopontin or poly-aspartic acid were used to induce mineralization of a collagen matrix by the polymer induced liquid precursor process. Although, similar mineralization was achieved, and the structural properties of the biomimetics were indistinguishable, osteoclasts were roughly 10-times more resorptive on the biomimetic-containing osteopontin [68]. Pure osteoclast cultures have been shown to resorb bone or hydroxyapatite substrates [32]. The increased specificity of mixed culture may come from osteoblastic cells interacting with the substrate and producing signals remove an osteoblastic block on osteoclastic resorption [68].

Integrin inhibitors have been shown to block bone resorption in vitro and in vivo [18,20,23,69] although they are insufficiently specific for osteoclasts to be useful as anti-resorptives. Likewise, interstitial collagenase inhibitors have the capacity to block resorption in vitro and in vivo, but are also not suitable to be used as systemically delivered anti-resorptives [27,67]. αVβ3 integrin is the major integrin on osteoclasts, but osteoclasts lacking β3, despite abnormalities, still resorb bone in vitro [26]. Resorption was less efficient and resulted in shallower pits. 

A second cell surface receptor implicated in triggering actin ring formation is CD44 [70]. αVβ3 and CD44 are differentially localized relative to the actin ring. αVβ3 is localized at the side of the actin ring where it co-localizes with proteins like vinculin and paxillin, which are found in focal adhesions in other cell types. CD44 is localized to the core region, the region where cortactin and Arp2/3 complex are also found [71]. This transmembrane protein is a receptor for hyaluronic acid, osteopontin, and collagens, and interacts with matrix metalloproteinases [72]. CD44 is involved in cell proliferation, cell differentiation, cell migration, angiogenesis, presentation of cytokines, chemokines, and growth factors to the corresponding receptors, and docking of proteases at the cell membrane, as well as in signaling for cell survival [73].

### 3.3. Unanswered Questions Regarding Mechanisms of Actin Ring Formation

A great deal is now known about the composition of actin rings. Perhaps the most intriguing component is cortactin, the pp60 c-src substrate that is required for actin rings and bone resorption and is at the heart of actin rings, and the podosomes and invadopodia of other cell types that invade tissue. Two questions arise with cortactin. In osteoclasts, and other podosomes forming cells, how does cortactin trigger the dynamics and complex structural organization of the actin cytoskeleton? Are there other components that shape the activity? Along that line, the cortactin seems most likely to work in conjunction with the Arp2/3 complex and n-WASP, but this has not been formally demonstrated in osteoclasts. Perhaps the dramatic structure and dynamics of actin rings is induced by one or more additional components that may give the polymerizing complex a unique characteristic that would allow it to be targeted therapeutically. 

It is possible that the actin isoform that is present in actin rings in biased toward beta or gamma actin. Although these actin isoforms are considered functionally interchangeable, increasing evidence has emerged suggesting that in cells specific actin structures are biased toward one or the other [74]. The biasing may be modulated by arginylation, which stimulates localization and assembly of beta actin, but degradation of gamma actin. The actin beta mRNA also has a “zip-code address” which localizes it to specific areas of some polarized cells [75]. The osteoclast would be a candidate to use that strategy. 

Both integrins and CD44 have been shown to be involved in controlling osteoclast activation. However, there is evidence for a mineral receptor. Osteoclast form actin rings on hydroxyapatite, but actin belts on plastic. There are various possibilities to explain this phenomenon, including an actual receptor for the hydroxyapatite, effects of calcium release, and the shape of the mineral nanocrystals. The ability to make increasingly bone-like biomimetics using defined components offers the possibility of dissecting the requirements for stimulating osteoclast activation [68].

### 3.4. Actin and the Formation of the Ruffle Membrane

The V-ATPase is required for osteoclastic bone resorption [3,4]. This enzyme is present in endosomes, lysosomes, and Golgi of all cells, at low levels and is responsible for the “housekeeping” acidification of these compartments [76]. It is found in a few cell types including osteoclasts and intercalated cells of the kidney, at much higher levels. These cells contain distinct subsets of V-ATPase; the housekeeping enzymes plus an additional subset that serves the specialized function of the cell type. In osteoclasts, the specialized V-ATPases are transported to the ruffled membranes of active osteoclasts and pump protons to acidify the resorption compartment [3,4].

**Figure 4:**
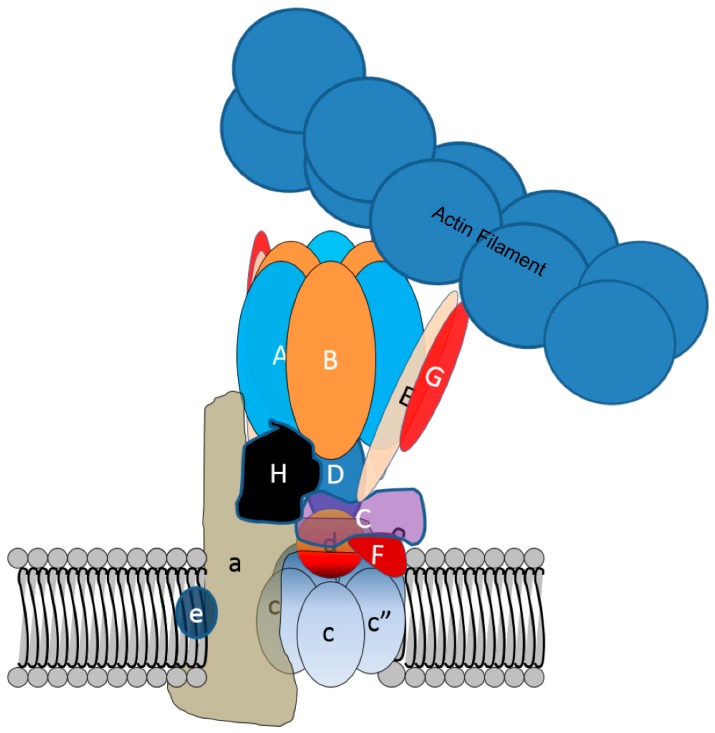
Despite a high affinity actin-binding site in the B-subunit of V-ATPase, normally V-ATPase does not bind microfilaments in cells. This is likely due to the actin binding site being covered by stator arms consisting of the E- and G-subunits. V-ATPase binds microfilaments through an actin binding site in the B2-subunit in osteoclasts when the actin binding site is available. Whether this is due to a change in position of the arm as depicted, or by the loss of one or more stator arms, is not known.

V-ATPases are multisubunit enzymes consisting of 13 different proteins and as many as 30 individual polypeptides [76]. This include the A, B, C, D, E, F, G, H, a, c, c”, d, and e subunits (Figure 4). In osteoclasts, the specialized ruffled membrane bound V-ATPases are marked by the presence of the a3-isoform of a-subunit, whereas housekeeping enzymes contain a1 and a2 [1]. Mutations in the gene coding for the a3-subunit in mice and humans lead to autosomal malignant osteopetrosis, a severe fatal form of bone disease [77]. 

As ruffled membranes form, and as the V-ATPases present in the ruffled membrane are reinternalized after a period of resorption, the V-ATPases colocalize with microfilaments. This is associated with increased binding of microfilaments by V-ATPases [78]. An actin binding site in the amino-terminal domain of the B2-subunit of V-ATPase plays a crucial role in the process [79]. This actin binding site is located between amino acids 29–73 of the B2-subunit and contains a profilin-like domain from amino acids 55–65 [80]. Profilin-like domain peptides bind actin weakly, and changes of specific amino acids within the profilin-like domain disrupts the capacity of the B-subunit to bind microfilaments. Although both isoforms of the mammalian B subunit, and B-subunits from V-ATPase as evolutionarily separate as that of the yeast [81] have high affinity (K_D_ 100–200 nM) microfilament-binding sites, most of the time in most cells V-ATPase is not bound to microfilaments. This is very likely because the actin binding sites on each of the three B-subunit per enzyme are typically blocked by contact with stator arms composed of E- and G-subunits [82]. The actin binding site could be exposed either by the release of one or more of the three EG dimers that function as stator arms or by a conformational change in one or more of the stators, as shown in Figure 4. In osteoclasts, binding can be triggered by blocking phosphatidylinositol 3-kinase activity [78]. 

**Figure 5:**
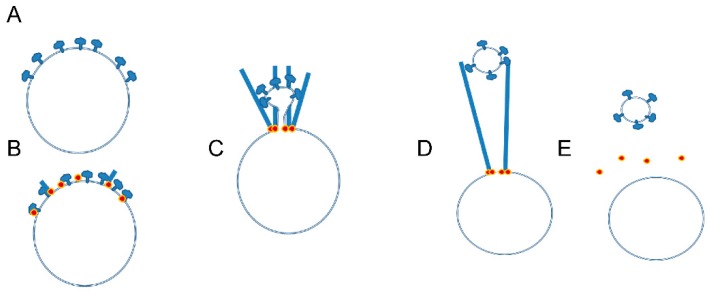
Model suggesting how V-ATPase binding to microfilaments can sort V-ATPase into a vesicle; (**A**,**B**) Arp2/3 complex or formin (shown as orange dot) is recruited to a vesicle at a site near V-ATPases and is activated to initiate actin polymerization with actin monomer adding on near the membrane, which is characteristic of actin polymerization in cells; (**C**) V-ATPase binds tightly to the actin filament at a specific location. As the actin filament elongates, the V-ATPase and associated membrane are drawn away from the vesicle membrane; (**D**) Eventually, the membrane tether breaks or is actively broken; (**E**) This leaves a mother vesicle depleted in V-ATPase and a daughter vesicle enriched in V-ATPase. This model is adapted from that of Carnell and colleagues [52]. The same basic mechanism could account for the internalization of V-ATPase from the ruffled plasma membrane.

Disruption of the actin-binding site in the B-subunit in osteoclasts blocked the transport of V-ATPase to ruffled membranes [79]. How might binding between V-ATPase and microfilaments be used to sort V-ATPases to subsets of vesicles or to and from the plasma membrane? One model to answer this question was developed based on a study of V-ATPase sorting in *Dictyostelium* is presented in Figure 5 [52]. The model hypothesizes Arp2/3 complex or a formin is recruited to site near the V-ATPase and initiates actin polymerization, as shown in Figure 5A,B. V-ATPase binds and stays bound to a specific actin molecule in the polymerizing filament. Since the filament is polymerizing at the vesicle membrane, as it elongates the V-ATPase is drawn away from the mother vesicle along with the associated membrane, as shown in Figure 5C. Eventually the membrane tether breaks, as shown in Figure 5D, and a new vesicle enriched in V-ATPase forms. The mother vesicle is at the same time depleted of V-ATPase, as shown in Figure 5E. In *Dictyostelium,* this sorting involved activation of the Arp2/3 complex by WASH [52].

We considered the actin binding site on the B2-subunit to be a therapeutic target, and hypothesized that a small molecule that binds the actin binding surface on B2 would compete with microfilaments for binding and disrupt osteoclast activity. A computational chemistry-based approach was taken to test this idea [83]. The B2-subunit was modeled, and the structure of the actin-binding site was determined. It was found to have two binding pockets. A DOCK program (DOCKv6), which uses geometric algorithms to predict the binding modes of small molecules, was used to assess 350,000 small molecules in the National Cancer Center’s small molecule library for the likelihood that they would bind to one or the other pocket. The 50 best candidates were obtained and screened for their ability to block binding between recombinant B2-subunit and microfilaments in an in vitro pelleting assay. Two molecules from that screen (one for each binding pocket) were selected for further study. Both inhibited the differentiation and bone resorptive activity of osteoclasts in cell culture experiments [83]. 

One of the two candidate small molecules was a fluoroquinolone antibiotic called enoxacin. Although it is still approved by the Federal Drug Administration of the United States and is used for the treatment of urinary tract infections in much of the world, it was taken from the market in the United States by its manufacturer. Because of this it had been extensively studied and was easily available in quantity for further study. Enoxacin inhibited osteoclast formation and bone resorption in vitro without inducing apoptosis [53]. An initial experiment testing whether enoxacin blocked bone loss due to ovariectomy in mice proved inconclusive. Although there was a trend for reduced bone loss it was not significant. This was likely due to the low bioavailability of enoxacin. 

To overcome this problem, the bisphosphonate derivative of enoxacin (bis-enoxacin) was constructed using an approach for targeting fluoroquinolone antibiotics to bone for the treatment of bone infections [84]. Bis-enoxacin, like enoxacin, blocked binding of recombinant B2-subunit to microfilaments and osteoclast formation and bone resorption in vitro [85]. Probably because it bound and was concentrated to the bone, bis-enoxacin blocked bone resorption at a concentration of less than 1 µM. 

Bis-enoxacin also effectively blocked bone resorption in vivo. It reduced osteoclast-dependent orthodontic tooth movement [82] and periodontal bone loss in rats [86], and blocked systemic oxidative stress associated with periodontal infection [87]. Recently, it was reported that bis-enoxacin inhibited bone loss associated with ovariectomy in a mouse model [88,89]. Strikingly, it also improved bone strength compared with zoledronate [88]. This was linked to increased glycoprotein content in bone in the animals treated with bis-enoxacin. These data suggest that in addition to acting as an anti-resorptive, bis-enoxacin also alters bone remodeling in such a manner as to result in the formation of bone with greater strength [88]. Part of the motivation in blocking osteoclast activity through the V-ATPase was that results from mice and humans with mutant V-ATPase subunits that suggested that inhibition of the osteoclast V-ATPase might be bone anabolic [90]. While the effects of bis-enoxacin do not support this idea, they do suggest that bis-enoxacin, in addition to being an anti-resorptive, alters bone remodeling in a way that may be therapeutically beneficial in a different manner than currently-used therapeutic agents. 

## 4. Summary and Perspectives

The actin-based cytoskeleton in osteoclasts in known to play two unusual roles. First, the actin cytoskeleton is organized to form an actin ring. The actin ring structure is based on podosomes (invadopodia), structures found in some other cell types. The podosomes in osteoclasts at this point seem to be compositionally similar or identical to podosomes in other cell types. The actin ring structure that the podosomes are organized into is unique to osteoclasts and presumably involves compositional and/or regulatory elements that are distinct from podosomes in other cells. 

Second, the interplay between the ruffled membrane component V-ATPase and microfilaments is unusual, but not unique. The ability to bind microfilaments exists in V-ATPase B-subunits in organisms as separate from mammals as yeast [1]. Instructively, the B1 isoform of B-subunit, which is not found in osteoclasts, binds microfilaments [80]. It is tempting to speculate that this interaction may be involved in the transport of large numbers of V-ATPases to the plasma membrane of proximal tubules and intercalated cells of the kidney [91]. In yeast, the actin binding site plays a role in conferring drug resistance, although the exact mechanism is not known [78]. Although the regulatory pathway leading to V-ATPase binding to microfilaments, and more generally the pathways to actin ring and ruffle membrane formation, are not fully understood, the idea of developing therapeutics based on inhibiting microfilament binding to V-ATPase has been demonstrated to be viable both in vitro and in vivo [85,86,87,88,92]. It seems very likely that more detailed understanding of the role of actin in osteoclastic bone resorption will lead to new types of therapeutic agents.
